# Secreted phospholipase A_2_ group X regulates peripheral sensitization to allergen

**DOI:** 10.1172/jci.insight.196711

**Published:** 2026-03-12

**Authors:** Ryan C. Murphy, Ying Lai, Yu-Hua Chow, Matt Liu, Brian D. Hondowicz, Dowon An, Marion Pepper, William A. Altemeier, Teal S. Hallstrand

**Affiliations:** 1 Division of Pulmonary, Critical Care, and Sleep Medicine,; 2 Center for Lung Biology, Department of Medicine,; 3 Department of Immunology, University of Washington, Seattle, Washington, USA.

**Keywords:** Immunology, Pulmonology, Allergy, Asthma, Eicosanoids

## Abstract

The molecular mechanisms responsible for the “atopic march” of allergic skin disease to allergic airway disease are incompletely understood. Secreted phospholipase A_2_ group X (sPLA_2_-X) is implicated in human asthma and modulates airway hyperresponsiveness (AHR) and inflammation in murine models of allergic asthma. We developed a complete proteolytic allergen model of dermal sensitization followed by airway challenge to mimic the “atopic march” and examined the role of sPLA_2_-X in regulating peripheral allergen sensitization, AHR, and airway inflammation. *Pla2g10^–/–^* mice receiving both house dust mite (HDM) peripheral sensitization and airway challenge had attenuated AHR relative to WT mice and lower airway eosinophils. Transgenic C57BL/6 *hPLA2G10* mice (only expressing the human sPLA_2_-X gene) receiving treatment with a small molecule inhibitor of sPLA_2_-X (ROC0929) during the dermal sensitization phase demonstrated attenuated AHR and a reduction HDM-specific tissue-resident memory CD4^+^ T cells in the lung. Thus, sPLA_2_-X acts as an endogenous adjuvant to facilitate allergic sensitization in the periphery, which leads to AHR and airway inflammation following inhalation of the allergen. These results provide proof of concept that inhibition of sensitization in the periphery with a sPLA_2_-X inhibitor modulates subsequent allergen-induced airway dysfunction.

## Abstract 2

Secreted phospholipase A2 group X plays a critical role in allergic sensitization and is a therapeutic target that modulates the development of allergic airway disease.

## Introduction

Phospholipase A_2_ (PLA_2_) enzymes serve as the initial rate-limiting step in endogenous eicosanoid synthesis and play key roles as regulators of inflammation. A class of these enzymes is also secreted into the extracellular space (secreted PLA_2_s [sPLA_2_]) and has additional functions, including the degradation of surfactant lipids and receptor-binding properties and are upregulated at sites of inflammation (1, 2). We have examined the sPLA_2_ family in asthma and found that sPLA_2_ group 10 (sPLA_2_-X) is the dominant sPLA_2_ that cleaves mammalian phospholipids in the airway fluid and that the levels of this protein in the airways are significantly increased in asthma and related to airway dysfunction in the form of airway hyperresponsiveness (AHR) (3–7). We further determined that airway epithelial cells and macrophages are key sources of sPLA_2_-X in the airways and that the levels of sPLA_2_-X are associated with dysregulated eicosanoid synthesis in the airways (5–7). Thus, sPLA_2_-X is implicated as a key regulator of endogenous eicosanoid synthesis and airway dysfunction in asthma.

Global deletion of the murine sPLA_2_-X gene (*Pla2g10*) in murine models of asthma results in protection from features of airway inflammation and airway dysfunction and lowers the levels of inflammatory eicosanoids to near basal levels (8, 9). This protection from airway inflammation and dysfunction occurs in a T cell–dependent model that requires an exogenous adjuvant (OVA-alum) (9), and also in a complete allergen model in which sensitization initially occurs in the airways in the absence of an exogenous adjuvant (8). Because prior work in humans and in mice indicate that sPLA_2_-X acts during both the sensitization and effector phases of asthma, we further examined the role of sPLA_2_-X as an adjuvant, showing that exogenous recombinant sPLA_2_-X and a similar sPLA_2_ from bee venom (bvPLA_2_) can act as an adjuvant, leading to sensitization and a type-2 dominant immune response with airway dysfunction upon challenge in the lungs (10). This adjuvant effect was dependent on phospholipid hydrolysis since a recombinant version of sPLA_2_-X that lacks enzymatic activity did not induce these changes (10).

In human asthma, sensitization to allergen often proceeds the development of asthma, with sensitization occurring in the upper airway or in the skin (11, 12). This “atopic march” from sensitization to airway dysfunction is rarely studied in model systems in the absence of an exogenous adjuvant. Given the function and endogenous expression of sPLA_2_-X in inflamed dermal tissue (13–16), we hypothesized that sensitization in the skin with a complete allergen such as that from house dust mite (HDM) would be attenuated when sPLA_2_-X was absent or inhibited, leading to an attenuated response to subsequent challenge. Thus, we developed a model of peripheral sensitization to HDM in the absence of an adjuvant followed by airway challenge and studied mice globally deficient in *Pla2g10* on both C57BL/6 and BALB/c backgrounds. Furthermore, we developed mice expressing human *PLA2G10* (*hPLA2G10*) in place of the murine enzyme and studied the effects of a human sPLA_2_-X inhibitor (ROC0929) that was administered concurrently with HDM peripheral sensitization, providing us with a window into the effects of sPLA_2_-X inhibition on peripheral allergen sensitization and development of AHR and airway inflammation following subsequent airway challenge.

## Results

### AHR and eosinophilic airway inflammation requires both peripheral sensitization and airway challenge.

**Figure 1:**
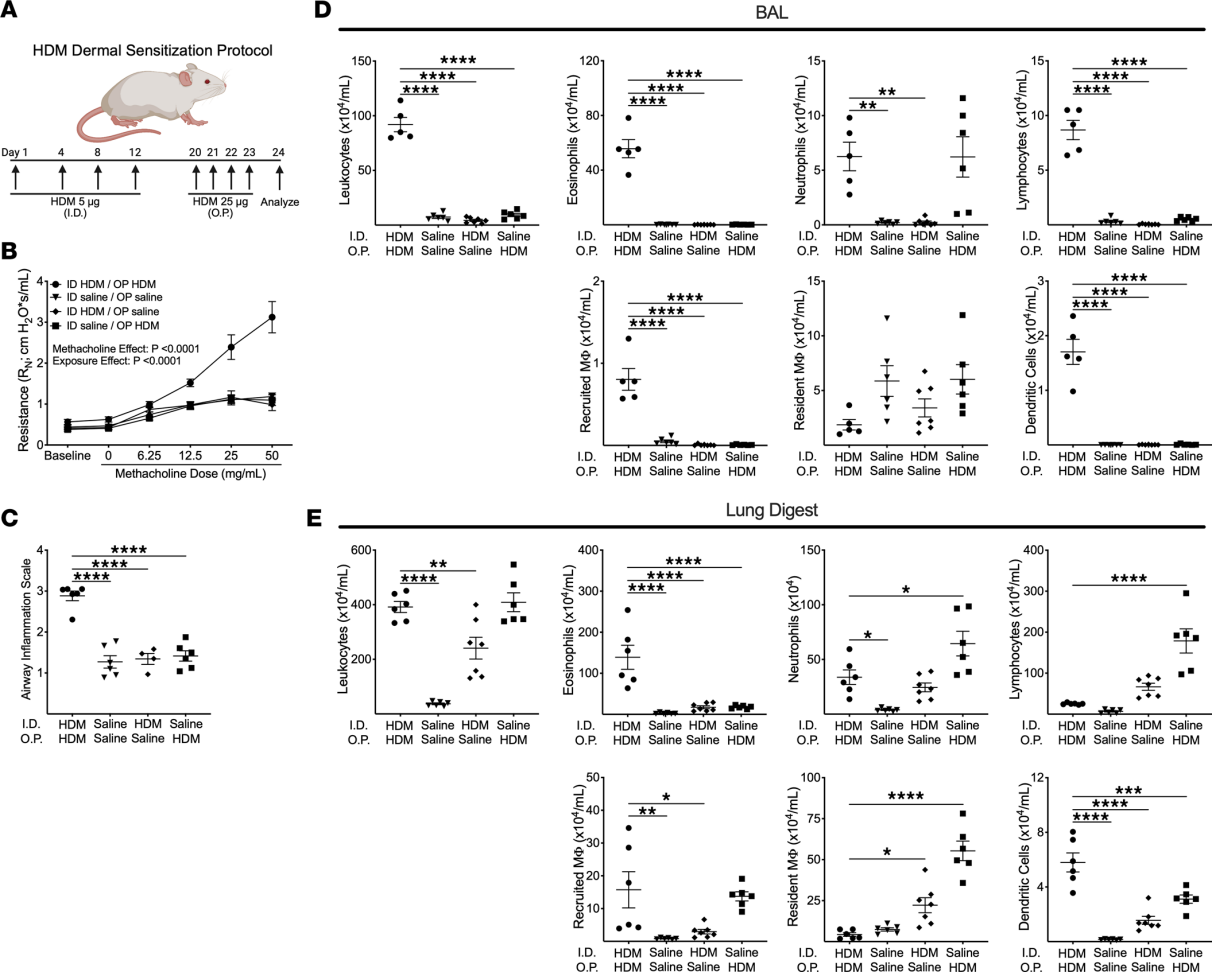
House dust mite (HDM) dermal sensitization and airway challenge model in WT BALB/c mice results in airway hyperresponsiveness (AHR) and airway inflammation. (**A**) HDM dermal sensitization and airway challenge protocol. (**B**) Measurement of AHR to increasing doses of methacholine (*n* = 6 intradermal [I.D.] HDM/oropharyngeal [O.P.] HDM, *n* = 6 I.D. saline/O.P. saline, *n* = 4 I.D. HDM/O.P. saline, *n* = 6 I.D. saline/O.P. HDM). Experiments were performed on 6 distinct days. *P* values are the result of a 2-way ANOVA. (**C**) Airway leukocyte infiltration was assessed in 10 large airways per mouse in H&E-stained sections of the right lung, and the mean value of histologic airway inflammation score was recorded. Leukocytes and individual leukocyte populations in bronchoalveolar lavage (BAL) fluid (**D**) and single-cell suspensions of cells from digested lung tissue (**E**) were characterized by spectral flow cytometry. MΦ, macrophages. *P < 0.05; **P < 0.01; ***P < 0.001; ****P < 0.0001 by 1-way ANOVA with multiple comparisons using the 2-stage step-up procedure of Benjamini, Krieger, and Yekutieli. All data are presented as mean ± SEM.

WT mice on a BALB/c background demonstrated significant AHR after receiving intradermal (i.d.) HDM followed by oropharyngeal challenge (o.p.) with HDM (Figure 1, A and B). However, there was no significant change in AHR in mice receiving saline either during the dermal sensitization phase or the airway challenge phase of the protocol in comparison to the control mice receiving both i.d. and o.p. saline. Relative to the other 3 conditions, mice receiving i.d. HDM/o.p. HDM demonstrated significantly higher airway cellular infiltration on histologic assessment (Figure 1C) as well as higher total leukocytes, eosinophils, lymphocytes, recruited macrophages, and dendritic cells in bronchoalveolar lavage (BAL) fluid (Figure 1D) and higher lung tissue eosinophils and dendritic cells (Figure 1E). There were higher numbers of lymphocytes and resident macrophages as well as similar numbers of recruited macrophages in the lungs of mice receiving i.d. saline/o.p. HDM relative to mice receiving i.d. HDM/o.p. HDM. Finally, lung neutrophils were significantly higher and BAL neutrophil counts were similarly increased in mice receiving i.d. saline/o.p. HDM relative to mice receiving i.d. HDM/o.p. HDM, suggesting that acute exposure to HDM extract can induce neutrophilic airway inflammation. We also found that WT mice on a C57BL/6 background have similar outcomes in this model, including a nonsignificant increase in airway resistance and higher BAL and lung eosinophilia (Supplemental Figure 1; supplemental material available online with this article; https://doi.org/10.1172/jci.insight.196711DS1).

### Pla2g10-deficient mice have attenuated AHR and airway inflammation following peripheral sensitization and challenge.

**Figure 2:**
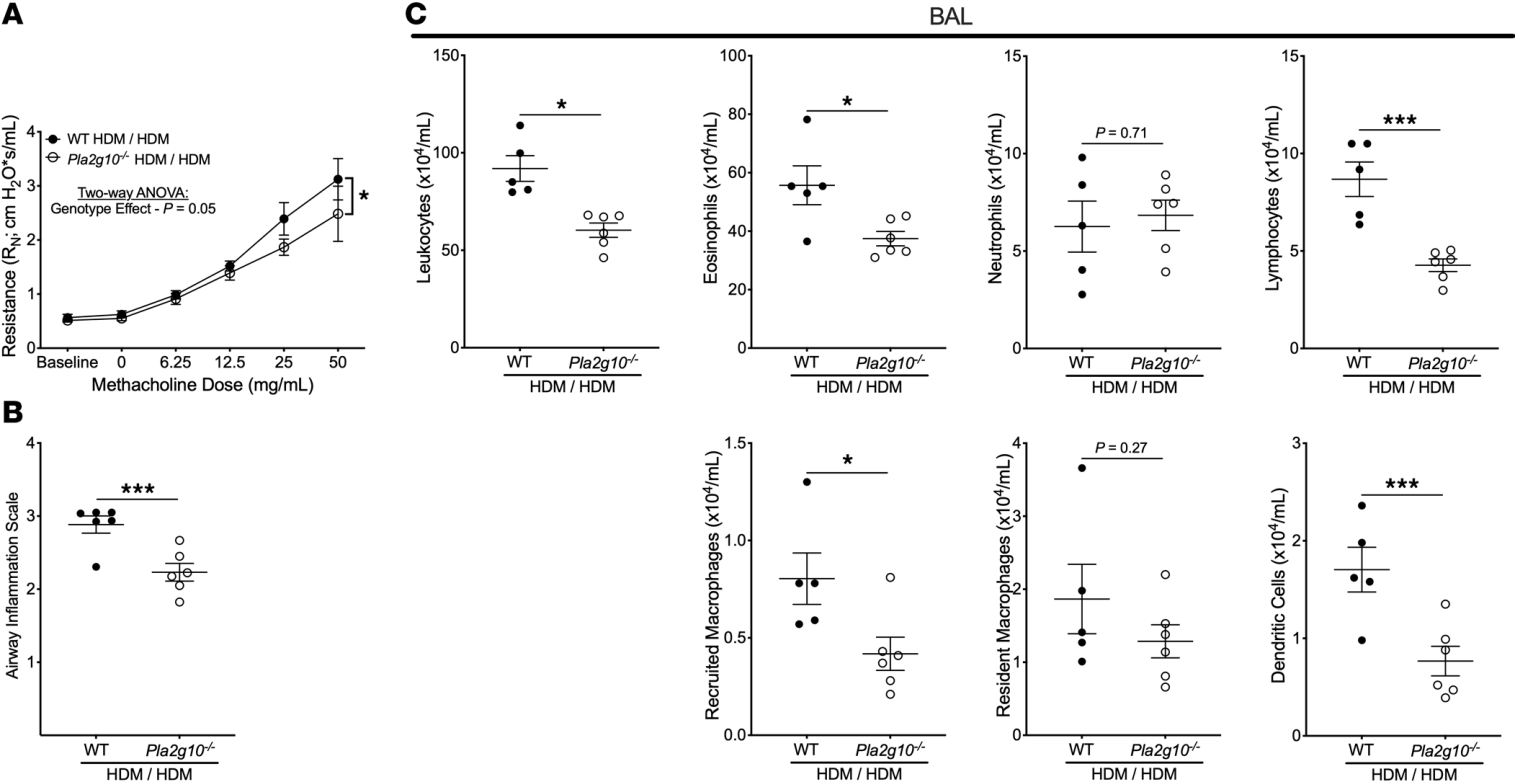
Mice globally deficient in *Pla2g10* have attenuated airway hyperresponsiveness (AHR) and airway inflammation in the house dust mite (HDM) dermal sensitization and airway challenge model. (**A**) Measurement of AHR to increasing doses of methacholine (*n* = 6 WT intradermal [I.D.] HDM/oropharyngeal [O.P.] HDM, *n* = 6 *Pla2g10^–/–^* I.D. HDM/O.P. HDM). Experiments were performed on 6 distinct days (concurrently performed experiments with WT mice shown in Figure 1). *P < 0.05 by 2-way ANOVA with multiple comparisons using the 2-stage step-up procedure of Benjamini, Krieger, and Yekutieli. (**B**) Airway leukocyte infiltration was assessed in 10 large airways per mouse in H&E-stained sections of the right lung, and the mean value of histologic airway inflammation score was recorded. Leukocytes and individual leukocyte populations in bronchoalveolar lavage (BAL) fluid (**C**) were characterized by spectral flow cytometry. *P < 0.05, ***P < 0.001 by 1-way ANOVA with multiple comparisons using the 2-stage step-up procedure of Benjamini, Krieger, and Yekutieli. All data are presented as mean ± SEM.

Mice that are globally deficient in *Pla2g10* (*Pla2g10^–/–^*) demonstrated attenuated AHR relative to WT mice after receiving i.d. HDM and o.p. HDM (Figure 2A), which corresponded with significantly reduced airway cellular infiltration (Figure 2B). *Pla2g10^–/–^* mice also had significantly fewer leukocytes, eosinophils, lymphocytes, recruited macrophages, and dendritic cells and similar neutrophils and resident macrophages in the airway lumen relative to WT mice (Figure 2C). We did not identify significant differences in total leukocytes or individual leukocyte populations in digested lung tissue between genotypes (Supplemental Figure 2), which could be explained by an inability to distinguish between the tissue and intravascular compartments of the lung that we have recently demonstrated can be evaluated with higher resolution using intravascular leukocyte labeling (17). There were significant increases in BAL levels of multiple inflammatory proteins (IL-1β, IL-4, IL-5, IL-6, IL-13, IL-18, TNF-α, IFN-γ, and thymic stromal lymphopoietin [TSLP]) in both WT and *Pla2g10^–/–^* mice receiving i.d. HDM/o.p. HDM relative to mice receiving i.d. saline/o.p. saline, but there were no significant differences between genotypes (Supplemental Figure 3).

### sPLA_2_-X is an endogenous adjuvant that facilitates peripheral allergic sensitization.

**Figure 3:**
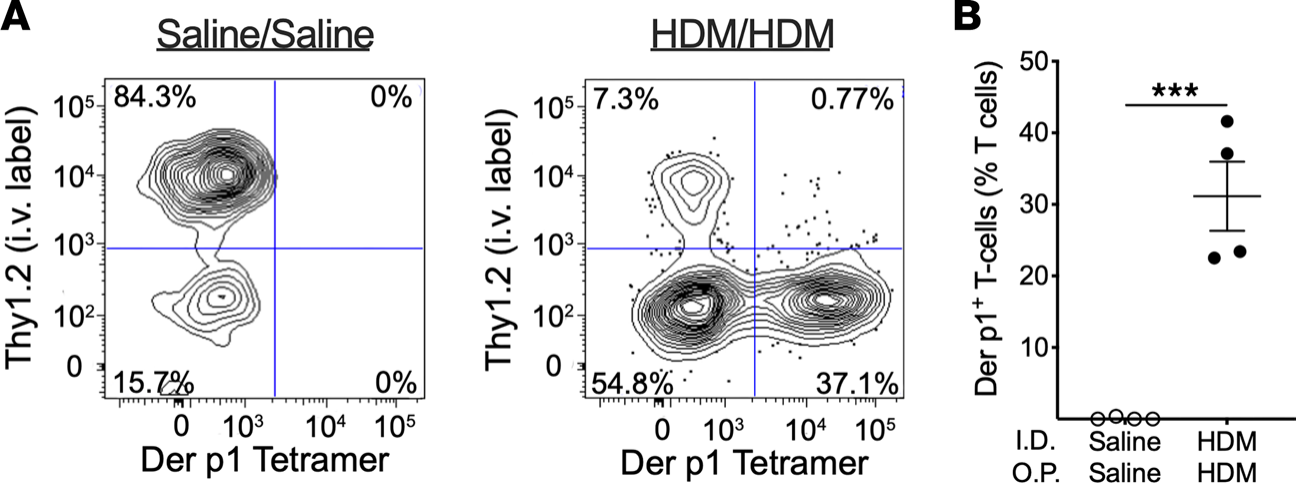
Characterization of *Dermatophagoides pteronyssinus* 1 (Der p1) CD4^+^ T cells in lung digest tissue. (**A**) Examples of flow cytometry gating strategy to identify lung tissue CD4^+^ T cells (Thy1.2^–^) expressing the major house dust mite (HDM) allergen epitope Der p1 (Der p1 tetramer^+^) for C57BL/6 mice receiving intradermal (I.D.) and oropharyngeal (O.P.) saline versus I.D. HDM and O.P. HDM. (**B**) Percentage of CD4^+^ T cells expressing Der p1 in WT C57BL/6 mice. Data are presented as mean ± SEM. *P < 0.05, ***P < 0.001 by unpaired 2-tailed *t* test.

**Figure 4:**
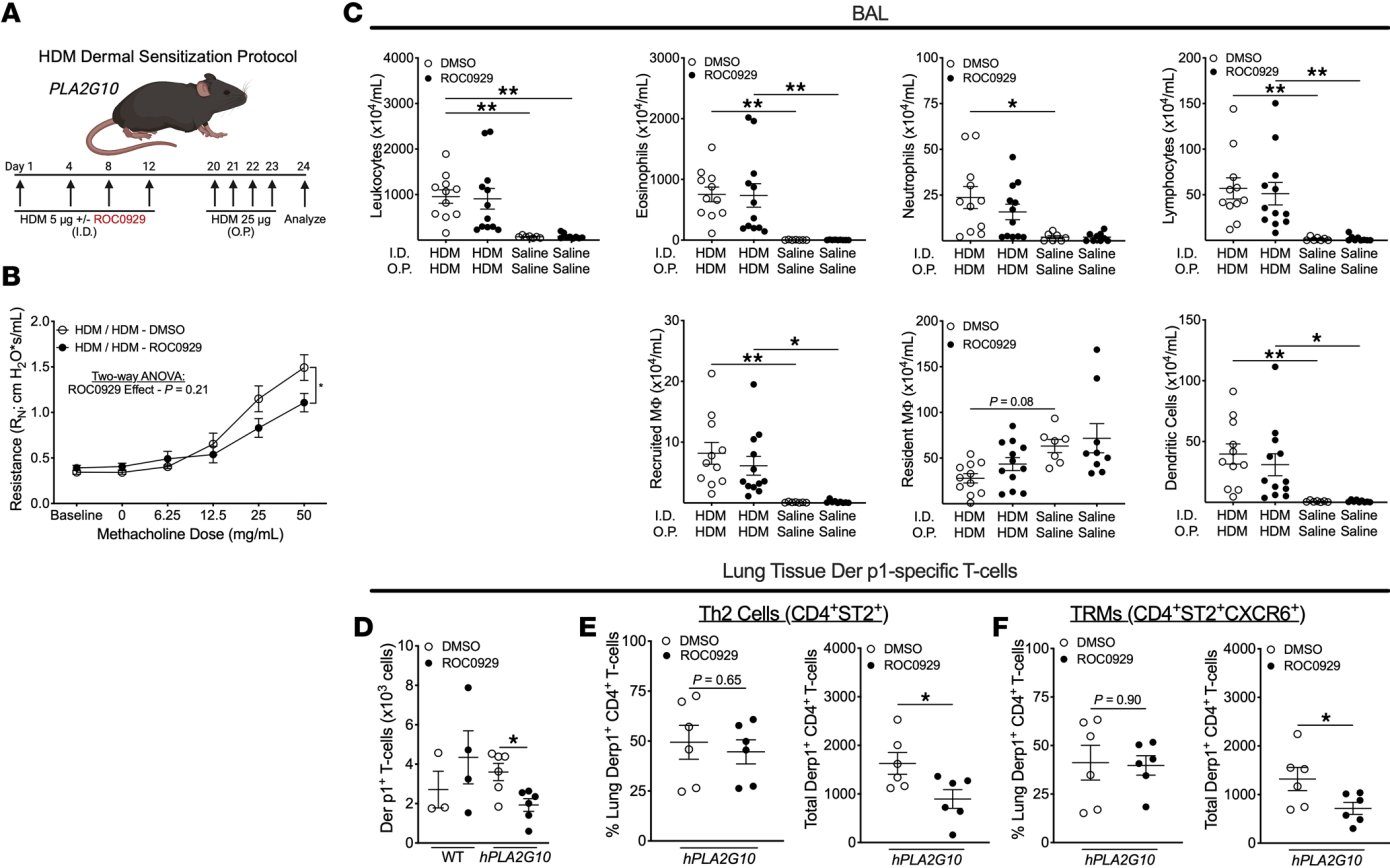
Treatment of mice expressing human *PLA2G10* with a sPLA_2_-X small molecule inhibitor during sensitization results in attenuated airway hyperresponsiveness (AHR). (**A**) House dust mite (HDM) dermal sensitization and airway challenge protocol with ROC0929 concurrently injected into the dermis during sensitization. (**B**) Measurement of AHR to increasing doses of methacholine (*n* = 6 WT intradermal [I.D.] HDM/oropharyngeal [O.P.] HDM, *n* = 6 *Pla2g10^–/–^* I.D. HDM/O.P. HDM). *P < 0.05 by 2-way ANOVA with multiple comparisons using the 2-stage step-up procedure of Benjamini, Krieger, and Yekutieli. (**C**) Leukocytes and individual leukocyte populations in bronchoalveolar lavage (BAL) fluid were characterized by spectral flow cytometry. MΦ, macrophages. *P < 0.05, **P < 0.01 by 1-way ANOVA with multiple comparisons using the 2-stage step-up procedure of Benjamini, Krieger, and Yekutieli. (**D**–**F**) Percentage of CD4^+^ T cells expressing Der p1 in C57BL/6 mice expressing *hPLA2G10* receiving either DMSO or ROC0929 (specific small molecule inhibitor of human sPLA_2_-X) during dermal sensitization. TRMs, tissue-resident memory cells. *P < 0.05 by unpaired 2-tailed *t* test. All data are presented as mean ± SEM.

Prior work by our lab demonstrated that sPLA_2_-X can promote peripheral sensitization to OVA via its enzymatic activity (10); therefore, we evaluated whether endogenous sPLA_2_-X was necessary for antigen sensitization. Because class II tetramers specific for the *Dermatophagoides* *pteronyssinus* 1 (Der p1) protein of HDM are only available for the C57BL/6 strain, we used this mouse strain to confirm recruitment of Der p1–specific T cells to the lung tissue in the dermal sensitization and o.p. challenge model. Mice sensitized and challenged with saline had T cells primarily within the intravascular compartment of the lung (Thy1.2^+^) and no significant Der p1–specific T cells (Der p1 tetramer^+^) (Figure 3A). In contrast, lung digest tissue obtained from mice with i.d. sensitization followed by o.p. challenge with HDM demonstrated a reduction in intravascular T lymphocytes (Thy1.2^+^) and a large increase in lung tissue Der p1 tetramer^+^ cells (Thy1.2^–^ and Der p1 tetramer^+^; Figure 3B). To evaluate the role of sPLA_2_-X in peripheral sensitization, we utilized transgenic C57BL/6 mice expressing *hPLA2G10* instead of murine *Pla2g10*. Mice received either the human sPLA_2_-X–specific inhibitor ROC0929 (4) or DMSO control via concurrent i.d. administration of HDM during sensitization (Figure 4A). ROC0929 treatment during peripheral sensitization resulted in attenuated AHR in response to methacholine (Figure 4B) but there was no significant difference in airway cellular inflammation following HDM sensitization and challenge between mice that received either DMSO or ROC0929 during sensitization (Figure 4C). Similarly, there were no differences in inflammatory cytokine levels between digested lung tissue obtained from mice receiving either DMSO or ROC0929 that underwent restimulation with HDM (Supplemental Figure 4). However, we did observe a significant reduction in Der p1–specific T cells recruited to the lung in *hPLA2G10* mice receiving ROC0929 during sensitization, indicating that peripheral inhibition of sPLA_2_-X resulted in attenuated allergic sensitization (Figure 4D). We confirmed that these Der p1–specific CD4^+^ Th2 cells are tissue-resident memory (TRM) cells (Figure 4, E and F), which have previously been shown to modulate AHR in murine models of allergic asthma (18).

## Discussion

Here, we examined the role of sPLA_2_-X in modulating allergic sensitization and subsequent airway dysfunction using a model system of peripheral sensitization followed by airway challenge of a complete proteolytic allergen (HDM). In this model of the atopic march, we confirm that the development of AHR and allergic airway inflammation requires both peripheral sensitization and airway challenge and that these features of airway dysfunction are reduced in mice globally lacking *Pla2g10*. We further demonstrate that allergic sensitization in the periphery can be attenuated with a human sPLA_2_-X inhibitor administered locally to transgenic mice expressing the *hPLA2G10* gene, which results in attenuation of AHR and diminished expansion of Der p1–specific TRM T cells in the lung tissue. Overall, we demonstrate that sPLA_2_-X modulates AHR and airway inflammation in murine models of allergic asthma but also uniquely characterize its role in peripheral allergen sensitization, which is a key component in the progression to allergic asthma. Further work is needed to more precisely identify the key cell populations and inflammatory mediators that are modulated by sPLA_2_-X function in the dermis but our findings suggest that therapeutic targeting of this enzyme in human populations may interrupt the atopic march to allergic airway disease.

Allergic sensitization in the upper airway and/or skin often proceeds the development of asthma (19, 20). While the overall relative risk of asthma is only modestly increased by atopy, allergic sensitization on an individual level increases the probability of developing asthma. The relationship between allergic sensitization and asthma is particularly critical when sensitization occurs in the skin with features of atopic dermatitis (21, 22) but the precise mechanisms of why some atopic individuals develop airway disease and others do not remains incompletely understood. Studies of peripheral sensitization before airway challenge have been predominantly conducted with an adjuvant in the peritoneum with an incomplete allergen (such as OVA-alum), and most studies of peripheral sensitization in the skin have used the exogenous administration of cytokines implicated in asthma such as TSLP and IL-33 (23, 24). We previously used this approach using recombinant versions of sPLA_2_-X to identify the function of sPLA_2_-X as an adjuvant in an OVA-alum model (10), but here extend these results to demonstrate the importance of sPLA_2_-X in allergic sensitization using a complete allergen. Common complete allergens such as HDM have properties that induce sensitization in the absence of an exogenous adjuvant and ongoing sensitization and allergen-induced responses during the effector phase in murine models, closely replicating human asthma. The property of complete allergens that mediate both development and maintenance of sensitization is the rationale behind therapeutic approaches to asthma, including allergen desensitization (25), the development of hypoallergens to facilitate desensitization (26), and IgE blockade as a therapeutic strategy in asthma (27). The complete allergen model of peripheral sensitization and subsequent airway challenge that we developed for this study is highly effective in inducing allergic airway dysfunction and we hope it can serve as a useful model system for investigators interested in further studying the mechanisms of the atopic march.

Our results indicate that sPLA_2_-X plays a critical role in peripheral allergic sensitization, as demonstrated by the reduction in allergen-specific T cells infiltrating the lung tissue in mice receiving sPLA_2_-X inhibition during dermal sensitization, which led to reduced AHR following subsequent airway allergen challenge. Relatively little is known about the sPLA_2_ family of enzymes in the skin, but multiple published studies support endogenous sPLA_2_-X activity in the skin or have characterized the effects of exogenous sPLA_2_-X in the dermis and epidermis. There are several ways in which sPLA_2_-X could mediate sensitization in the skin, including lipid mediator formation and receptor binding properties. The PLA_2_ receptor (PLA2R1), which is expressed as either a transmembrane or soluble form, is a paralog of the mannose receptor, but in mice it appears to act primarily as a mechanism to attenuate sPLA_2_-X–mediated effects by binding to sPLA_2_-X and other sPLA_2_s (28). The mechanism of sensitization is almost certainly due to the actions of sPLA_2_-X on cells expressing 5-lipoxygenase in the dermal region leading to the formation of cysteinyl leukotrienes (CysLT) and other lipid mediators. Prior work on endogenous sensation to the complete allergen HDM demonstrates that CysLTs acting through the CysLT1 receptor and glycan binding are necessary for sensitization in this context (29, 30). Our prior work in this area demonstrates that sPLA_2_-X can act as an effective adjuvant when administered exogenously, and that this effect is dependent on the phospholipid hydrolysis function of the enzyme, suggesting that the formation of lipid mediators is the primary mechanism leading to sensitization (10). This suggests that sPLA_2_-X is generated in response to inflammation in the dermis and acts on cells expressing the leukotriene synthetic pathway, include eosinophils, mast cells, Langerhans cells, and dendritic cells in the skin. In this regard, in addition to Langerhans cells that are known to reside in this region, mast cells are known to infiltrate areas of inflamed skin (31, 32). Additional work in this area is needed regarding the precise source of sPLA_2_-X, the target cells, and the modulation of membrane-bound and free PLA2R1.

In summary, we developed a model of peripheral sensitization to HDM in the absence of an adjuvant followed by airway challenge, revealing that significant inflammation and airway dysfunction only occurred when short-term airway challenge was preceded by dermal sensitization. We demonstrated this response in both C57BL/6 and BALB/c mice but found that BALB/c mice had more AHR in this context. In BALB/c mice with a global *Pla2g10* deletion, we demonstrate decreased eosinophilic airway inflammation and AHR to inhaled methacholine following sensitization and challenge. However, we have previously shown that sPLA_2_-X has a dual role in allergic airways disease through modulating allergen sensitization and airway inflammation through innate and adaptive immune mechanisms, which can each contribute to the attenuated AHR seen in *Pla2g10*-deficient mice in this study and our prior model of HDM airway sensitization and challenge (8). Here, we further clarify the role of sPLA_2_-X in peripheral allergen sensitization by leveraging our model system that separates the sensitization and effector phases of the allergic response. Using a mouse that expresses *hPLA2G10* in place of the murine enzyme, we demonstrate that a human sPLA_2_-X inhibitor (ROC0929) administered with the sensitization doses of HDM significantly reduced sensitization (as evidenced by a reduction in Der p1 tetramer–expressing T cells), and reduced associated airway dysfunction after challenge with HDM allergen. However, it is notable that this attenuation of AHR was not associated with significant differences in immune cell infiltration of the airway lumen or lung tissue or inflammatory cytokine production by lung tissue immune cells restimulated with allergen. Although lung-resident allergen-specific T cells are sufficient to modulate AHR, the precise mechanism by which this occurs is not entirely understood (18). Overall, these findings demonstrate a specific role for sPLA_2_-X during endogenous sensitization in the absence of an exogenous adjuvant and that inhibition of such sensitization through a sPLA_2_-X–specific inhibitor could have therapeutic efficacy by blocking human sPLA_2_-X in the dermis.

## Methods

### Sex as a biological variable.

Our study exclusively studied female mice due to our interest in AHR to methacholine as a critical endpoint for our study. Female mice tend to exhibit more AHR relative to male mice, and thus female mice were utilized exclusively to reduce heterogeneity. It is unknown whether the findings are relevant for male mice.

### Mice.

WT BALB/c mice and WT C57BL/6 mice were obtained from The Jackson Laboratory. *Pla2g10^–/–^* mice were originally developed on a mixed 129SvEv^Brd^/C57BL/6J background and characterized as previously described (9). Mice were subsequently backcrossed onto the BALB/cJ background for 10 generations. Transgenic *hPLA2G10* mice (on a C57BL/6 background) were developed by inserting cDNA coding for *hPLA2G10* into exon 1 of the mouse *Pla2g10* gene, as previously reported (33). Animals were maintained under specific pathogen–free conditions at the University of Washington and had ad libitum access to food and water. Female mice between 6 and 12 weeks old were used in all experiments to reduce heterogeneity.

### HDM dermal sensitization and airway challenge model of allergic airway inflammation.

Mice received i.d. injections with 5 μg/injection of HDM extract (catalog RMB84M, lot 275533, Greer Laboratories) or saline on days 1, 4, 8, and 12. To perform i.d. injections, fur was stripped from the base of the tail and 50 μL was injected i.d. In addition to receiving HDM, *hPLA2G10* mice simultaneously received 50 μL i.d. of either DMSO or 100 nM ROC0929 in DMSO. Mice subsequently received o.p. administration of either HDM extract (25 μg/day) or saline on days 20, 21, 22, and 23 followed by collection on day 24 (Figure 1A and Figure 4A). This resulted in 4 experimental conditions per mouse: (a) i.d. HDM/o.p. HDM; (b) i.d. saline/o.p. saline; (c) i.d. HDM/o.p. saline; (d) i.d. saline/o.p. HDM. On day 24, mice were intubated and ventilated using a flexiVent small animal ventilator (SCIREQ Inc). Lung function was measured following increasing doses of methacholine (0, 6.25, 12.5, 25, and 50 mg/mL) and dynamic resistance was calculated using the single forced oscillation technique.

### Assessment of airway inflammation.

BAL was performed and the left lung was removed and processed into a single-cell suspension, as previously described (10). Total BAL and lung-digest cell counts were determined using a Cellometer Auto 2000 (Nexcelom Bioscience). Cells from BAL and digested lung were aliquoted for cell differential analysis via multicolor flow cytometry. BAL supernatant was stored at –80°C with 1× protease inhibitor cocktail (Pierce).

### Histologic assessment of airway inflammation.

The right lung was fixed for 24 hours in 10% formalin and embedded in paraffin. Sections (4 μm) were stained with hematoxylin and eosin (H&E) to assess inflammatory cell infiltrate. Slides were assessed by a scientist blinded to condition or genotype. Ten airways were assessed for each mouse using Visiopharm and were graded on a scale of 0–4, with 0 defined as absent and 4 as severe cellular infiltration. The histologic airway inflammation score was recorded as the mean score.

### Spectral flow cytometry of leukocyte populations.

Spectral flow cytometry was performed to identify leukocyte populations among BAL and lung-digest cells using a 6-fluorochrome panel for cell staining and gating strategy, as previously described (17): anti-CD11c (clone N418, BioLegend), anti–Siglec-F (clone E50-2440, BD Biosciences), anti–Ly-6G (clone RB6-8C5, eBiosciences), anti-CD45 (clone 30-F11, BioLegend), anti-CD3e (clone 145-2C11, BioLegend), and anti-MHCII (clone 2G9, BD Biosciences). Cells were analyzed using a 5-laser Aurora spectral flow cytometer (Cytek Biosciences) and data were analyzed using FlowJo software (TreeStar). Leukocyte populations (CD45^+^ cells) were defined as follows: neutrophils (CD3e^–^MHCII^lo^CD11c^–^Siglec-F^–^Ly-6G^+^), T lymphocytes (CD3e^+^), eosinophils (CD3e^–^MHCII^lo^CD11c^int^Siglec-F^+^), resident macrophages (CD3e^–^MHCII^lo^CD11c^hi^Siglec-F^+^), recruited macrophages (CD3e^–^MHCII^hi^CD11c^–^Siglec-F^–^), and dendritic cells (CD3e^–^MHCII^hi^CD11c^+^Siglec-F^–^).

### Antigen restimulation of cultured lung leukocytes.

For HDM restimulation of isolated lung leukocytes, 2 × 10^5^ cells were plated in each well of a 96-well tissue culture plate. Cells were stimulated with 100 μg/mL HDM extract or with saline as a control. Following stimulation for 24 hours, the cell culture supernatant was isolated and stored at –80°C with 1× protease inhibitor cocktail (Pierce). Cell culture supernatant was later analyzed for cytokine levels via ELISA.

### Cytokine and immunoglobin analysis by ELISA.

Detection of murine cytokines (IL-1β, IL-4, IL-5, IL-6, IL-13, TNF-α, IFN-γ, and TSLP) from BAL supernatant and from supernatant obtained from HDM-restimulated lung leukocytes was performed using a custom 13-plex ProcartaPlex Immunoassay (Thermo Fisher Scientific). Plates were analyzed using a Luminex 200 Instrument System.

### Assessment of Der p1–specific T cell populations.

To detect Der p1–specific T cell populations, biotinylated I-Ab molecules containing the covalently attached Der p1 114–124 (SNYCQIYPPNV) or Der p1 117–127 (CQIYPPNVNKI) epitope (nonamer core plus 2 N-terminal flanking amino acids) were made as previously described (34). Monomers were tetramerized with streptavidin-APC (Prozyme). Mice were injected i.v. with 1 mg of anti-Thy1.2–BUV 395 (clone 53-2.1, BD Biosciences) 3 minutes prior to euthanasia to label intravascular lymphocytes. Lungs were harvested following euthanasia and diced with scissors in complete tissue culture media (DMEM, 2-ME, P/S, L-glutamine, HEPES, and 10% fetal calf serum). The chopped lung fragments were then forced though a 0.45 mm strainer with the back of a 3 cc syringe plunger and the strainer was rinsed with culture media. The single-cell suspensions were stained with Der p1:I-Ab tetramer conjugated to APC for 60 minutes in the dark at room temperature. The lung cells were then washed and incubated with anti-APC microbeads (Miltenyi Biotec) for 30 minutes. Tetramer^+^ T cells were enriched over an LS column. The bound fraction was then collected, spun, washed, stained with anti-ST2 (biotinylated, clone DJ8, MD Bioproducts; Streptavidin BV605, BD Biosciences) and anti-CXCR6 antibodies (PE/Dazzle 594, clone SA051D1, BioLegend) and evaluated on a BD Symphony flow cytometer. The data were analyzed in FlowJo to identify live, single cells and differentiate between intravascular (Thy1.2^+^) and tissue (Thy1.2^–^) Der p1–specific T-lymphocytes (Der p1 tetramer^+^).

### Statistics.

Statistical methods are explicitly discussed within individual figure legends. A *P* value of less than 0.05 was considered significant.

### Data availability.

Values for all data points shown in graphs of the main and supplemental figures are presented in the Supporting Data Values file.

### Study approval.

The University of Washington Institutional Animal Care and Use Committee approved all animal studies.

## Author contributions

RCM, WAA, and TSH designed the studies. RCM, YL, YHC, ML, BDH, and DA conducted the experiments and acquired the data. RCM, YHC, BDH, MP, WAA, and TSH analyzed the data. RCM, WAA, and TSH wrote, edited, and revised the manuscript. All authors reviewed and approved the manuscript prior to submission.

## Conflict of interest

The authors have declared that no conflict of interest exists.

## Funding support

This work is the result of NIH funding, in whole or in part, and is subject to the NIH Public Access Policy. Through acceptance of this federal funding, the NIH has been given a right to make the work publicly available in PubMed Central.

- NIH grants K24 AI130263 and R01 HL153979 (to TSH).
- NIH grant R21 AI152488 (to WAA, TSH).

## Supplementary Material

Supplemental data

Supporting data values

## Version 1 03/12/2026

In-Press Preview

## Version 2 05/08/2026

Electronic publication

## References

[1] Murakami M (2020). Updating phospholipase A_2_ biology. Biomolecules.

[2] Nolin JD (2019). Function of secreted phospholipase A_2_ group-X in asthma and allergic disease. Biochim Biophys Acta Mol Cell Biol Lipids.

[3] Murphy RC (2021). Exercise-induced alterations in phospholipid hydrolysis, airway surfactant, and eicosanoids and their role in airway hyperresponsiveness in asthma. Am J Physiol Lung Cell Mol Physiol.

[4] Hallstrand TS (2016). Endogenous secreted phospholipase A2 group X regulates cysteinyl leukotrienes synthesis by human eosinophils. J Allergy Clin Immunol.

[5] Hallstrand TS (2013). Regulation and function of epithelial secreted phospholipase A_2_ group X in asthma. Am J Respir Crit Care Med.

[6] Hallstrand TS (2011). Relationship between levels of secreted phospholipase A_2_ groups IIA and X in the airways and asthma severity. Clin Exp Allergy.

[7] Hallstrand TS (2007). Secreted phospholipase A_2_ group X overexpression in asthma and bronchial hyperresponsiveness. Am J Respir Crit Care Med.

[8] Nolin JD (2017). Secreted PLA2 group X orchestrates innate and adaptive immune responses to inhaled allergen. JCI Insight.

[9] (2007). Importance of group X-secreted phospholipase A_2_ in allergen-induced airway inflammation and remodeling in a mouse asthma model. J Exp Med.

[10] Ogden HL (2020). Secreted phospholipase A_2_ group X acts as an adjuvant for type 2 inflammation, leading to an allergen-specific immune response in the lung. J Immunol.

[11] Akar-Ghibril N (2020). Allergic endotypes and phenotypes of asthma. J Allergy Clin Immunol Pract.

[12] Bantz SK (2014). The atopic march: progression from atopic dermatitis to allergic rhinitis and asthma. J Clin Cell Immunol.

[13] Murakami M (2018). Phospholipase A_2_ in skin biology: new insights from gene-manipulated mice and lipidomics. Inflamm Regen.

[14] Yamamoto K (2011). Hair follicular expression and function of group X secreted phospholipase A_2_ in mouse skin. J Biol Chem.

[15] Ingber A (2007). A novel treatment of contact dermatitis by topical application of phospholipase A_2_ inhibitor: a double-blind placebo-controlled pilot study. Int J Immunopathol Pharmacol.

[16] Scott GA (2006). sPLA2-X stimulates cutaneous melanocyte dendricity and pigmentation through a lysophosphatidylcholine-dependent mechanism. J Invest Dermatol.

[17] Chow YH (2023). Intravascular leukocyte labeling refines the distribution of myeloid cells in the lung in models of allergen-induced airway inflammation. Immunohorizons.

[18] Hondowicz BD (2016). Interleukin-2-dependent allergen-specific tissue-resident memory cells drive asthma. Immunity.

[19] Dharmage SC (2022). Revisiting the atopic march current evidence. Am J Respir Crit Care Med.

[20] Hill DA, Spergel JM (2018). The atopic march: critical evidence and clinical relevance. Ann Allergy Asthma Immunol.

[21] Dijoux E (2023). Allergic sensitization driving immune phenotyping and disease severity in a mouse model of asthma. Allergy Asthma Immunol Res.

[22] Ravnborg N (2021). Prevalence of asthma in patients with atopic dermatitis: a systematic review and meta-analysis. J Am Acad Dermatol.

[23] Han H, Ziegler SF (2017). Intradermal administration of IL-33 induces allergic airway inflammation. Sci Rep.

[24] Han H (2012). Thymic stromal lymphopoietin (TSLP)-mediated dermal inflammation aggravates experimental asthma. Mucosal Immunol.

[25] Woehlk C (2022). Allergen immunotherapy effectively reduces the risk of exacerbations and lower respiratory tract infections in both seasonal and perennial allergic asthma: a nationwide epidemiological study. Eur Respir J.

[26] Glesner J (2019). A human IgE antibody binding site on Der p 2 for the design of a recombinant allergen for immunotherapy. J Immunol.

[27] Pelaia G (2017). Targeted therapy in severe asthma today: focus on immunoglobulin E. Drug Des Devel Ther.

[28] Nolin JD (2016). Identification of epithelial phospholipase A_2_ receptor 1 as a potential target in asthma. Am J Respir Cell Mol Biol.

[29] Barrett NA (2011). Dectin-2 mediates Th2 immunity through the generation of cysteinyl leukotrienes. J Exp Med.

[30] Barrett NA (2009). Dectin-2 recognition of house dust mite triggers cysteinyl leukotriene generation by dendritic cells. J Immunol.

[31] Keith YH (2023). Mast cells in type 2 skin inflammation: Maintenance and function. Eur J Immunol.

[32] Harvima IT, Nilsson G (2011). Mast cells as regulators of skin inflammation and immunity. Acta Derm Venereol.

[33] (2011). Blockade of human group X secreted phospholipase A_2_ (GX-sPLA2)-induced airway inflammation and hyperresponsiveness in a mouse asthma model by a selective GX-sPLA2 inhibitor. J Biol Chem.

[34] Moon JJ (2007). Naive CD4(+) T cell frequency varies for different epitopes and predicts repertoire diversity and response magnitude. Immunity.

