# Supplementary material for: Secreted phospholipase A2 group X regulates peripheral sensitization to allergen
Source: JCI Insight. 2026 Mar 12;11(9):e196711. doi: 10.1172/jci.insight.196711 (PMC13232007; doi:10.1172/jci.insight.196711)
Supplement: Supplemental data [file jciinsight-11-196711-s130.pdf]

Online Supplement

Secreted Phospholipase A_2 Group X Regulates Peripheral Sensitization to Allergen

Ryan C. Murphy[1,2], Ying Lai[1,2], Yu-Hua Chow[1,2], Matt Liu[1,2], Brian D. Hondowicz[3], Dowon An[1,2], Marion Pepper[3], William A. Altemeier[1,2], & Teal S. Hallstrand[1,2]

[1]Division of Pulmonary, Critical Care, and Sleep Medicine, [2]Center for Lung Biology, Department of Medicine, [3]Department of Immunology, University of Washington, Seattle, Washington

Corresponding Author:

Teal S. Hallstrand, MD, MPH

Division of Pulmonary, Critical Care, and Sleep Medicine

Center for Lung Biology, University of Washington

Box 358052, 850 Republican Street Seattle, 98109-4714.

Phone 206-221-0523, Fax 206-221-0739, Email: tealh@uw.edu

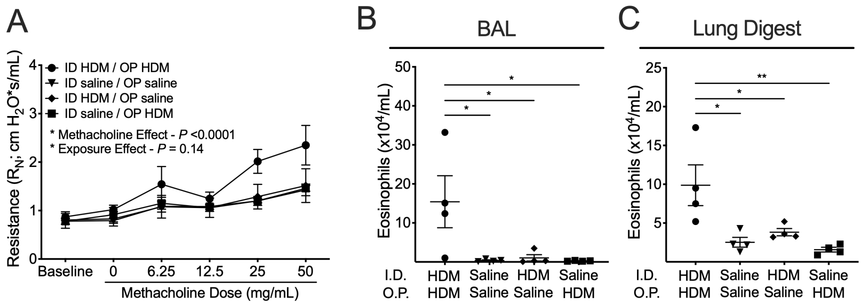

Supplemental Figure 1. House dust mite (HDM) dermal sensitization and airway challenge model in WT C57BL/6 mice results in airway hyperresponsiveness (AHR) and airway inflammation. (*A*) Measurement of AHR to increasing doses of methacholine (n = 4 intradermal (I.D.) HDM / oropharyngeal (O.P.) HDM, n = 4 I.D. saline / O.P. saline, n = 3 I.D. HDM / O.P. saline, n = 4 I.D. saline / O.P. HDM). Mean values are shown with error bars representing the standard error of the mean. *P* values are the result of a 2-way ANOVA. Eosinophils in bronchoalveolar lavage (BAL) fluid (*B*) and single cell suspensions of cells from digested lung tissue (*C*) were characterized by spectral flow cytometry. Mean values are shown with error bars representing the standard error of the mean. *P* values are the result of a 1-way ANOVA with multiple comparisons using the two-stage step-up procedure of Benjamini, Krieger, and Yekutieli. * indicates a *P* value <0.05 and ** indicates a *P* value <0.01.

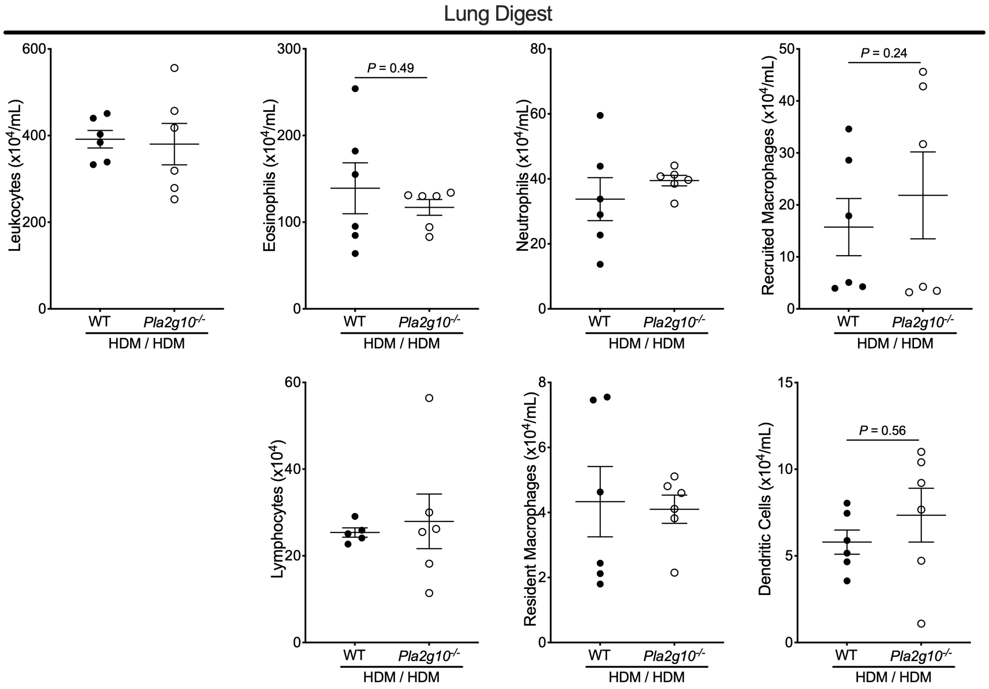

Supplemental Figure 2. Leukocytes and individual leukocyte populations in single cell suspensions of cells from digested lung tissue were characterized by spectral flow cytometry. Mean values are shown with error bars representing the standard error of the mean. *P* values are the result of a 1-way ANOVA with multiple comparisons using the two-stage step-up procedure of Benjamini, Krieger, and Yekutieli

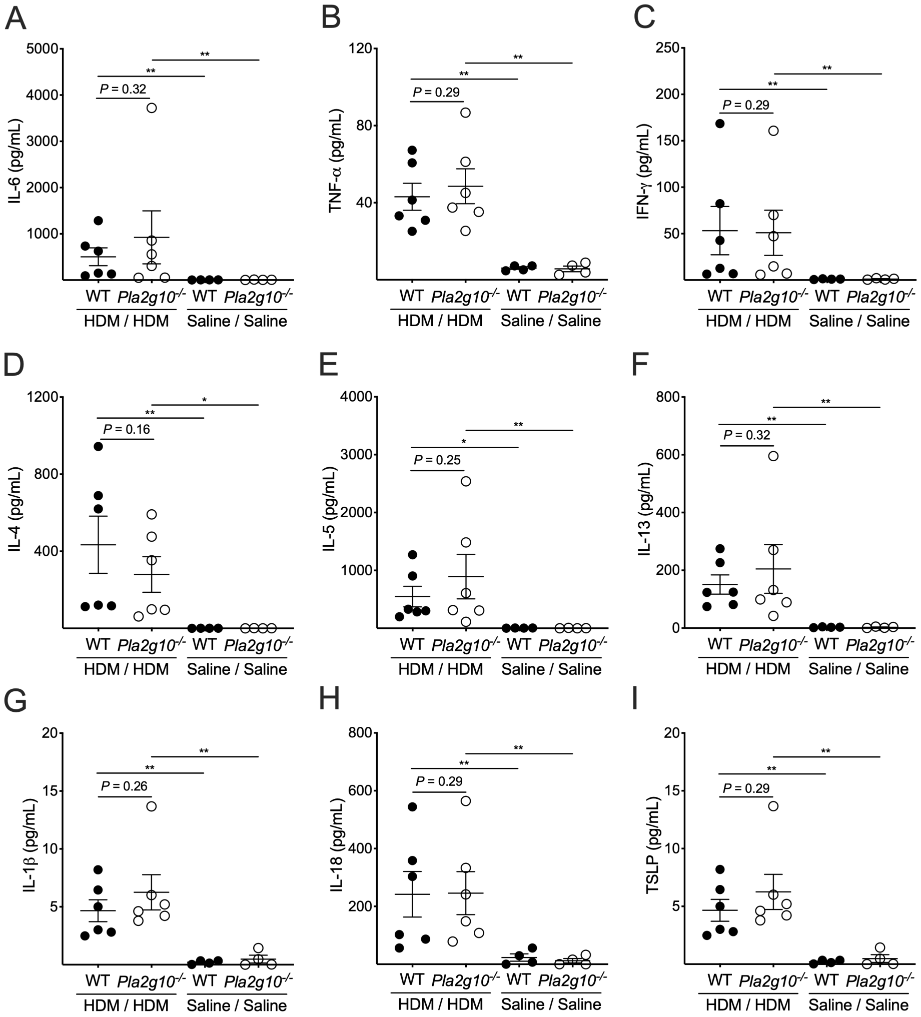

Supplemental Figure 3. Measurements of protein levels in bronchoalveolar lavage (BAL) fluid from WT and *Pla2g10*$^{-/-}$ BALB/c mice. *P* values are the results of Kruskal-Wallis test with multiple comparisons using the two-stage step-up procedure of Benjamini, Krieger, and Yekutieli. * indicates a *P* value <0.05 and ** indicates a *P* value <0.01. HDM (house dust mite).

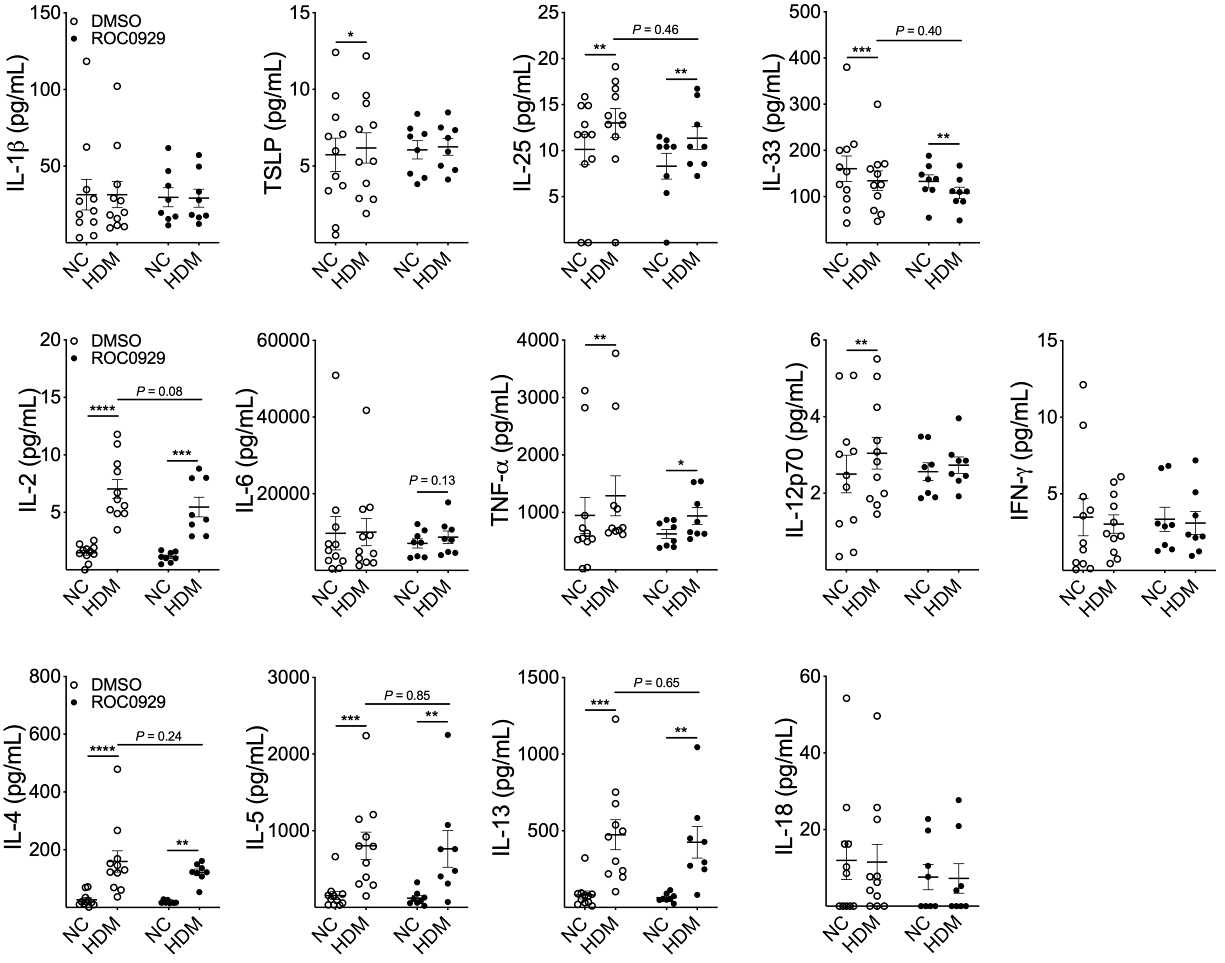

Supplemental Figure 4. Measurements of protein levels in the supernatant of lung digest cells following restimulation with either house dust mite (HDM) or saline (negative control, NC). Lung digest cells were obtained from *PLA2G10* C57BL/6 mice undergoing intradermal HDM sensitization and oropharyngeal HDM challenge that received treatment with either DMSO or ROC0929 during intradermal sensitization. *P* values are the result of a 2-way ANOVA with uncorrected Fisher's LSD test. * indicates a *P* value <0.05, ** indicates a *P* value <0.01, *** indicates a *P* value <0.001, and **** indicates a *P* value <0.0001.
